# Necrobiosis lipoidica

**DOI:** 10.3402/jchimp.v3i3-4.22627

**Published:** 2013-12-17

**Authors:** Ranjan Pathak, Paras Karmacharya, Madan Raj Aryal, Karen E. Smith-Coleman

**Affiliations:** 1 Department of Internal Medicine, Reading Health System, West Reading, PA, USA; 2 RPS Endocrinology Diabetes and Metabolism Center, Wyomissing, PA, USA

A47-year-old woman reported an 18-month-old pretibial rash on her left shin during a routine office visit. She had a history of type 1 diabetes mellitus (DM) and has been on insulin therapy for the past 13 years. She did not complain of any itching, pain, or bleeding from the rash. On examination, an 8×3 cm elongated indurated plaque with telangiectases, central atrophy, and yellow pigmentation was noted. Reddishbrown pigmentation was present peripherally along the margins. No ulcerations were present. Patient was diagnosed to have necrobiosis lipoidica on the basis of history and typical features on examination.

**Fig. 1:**
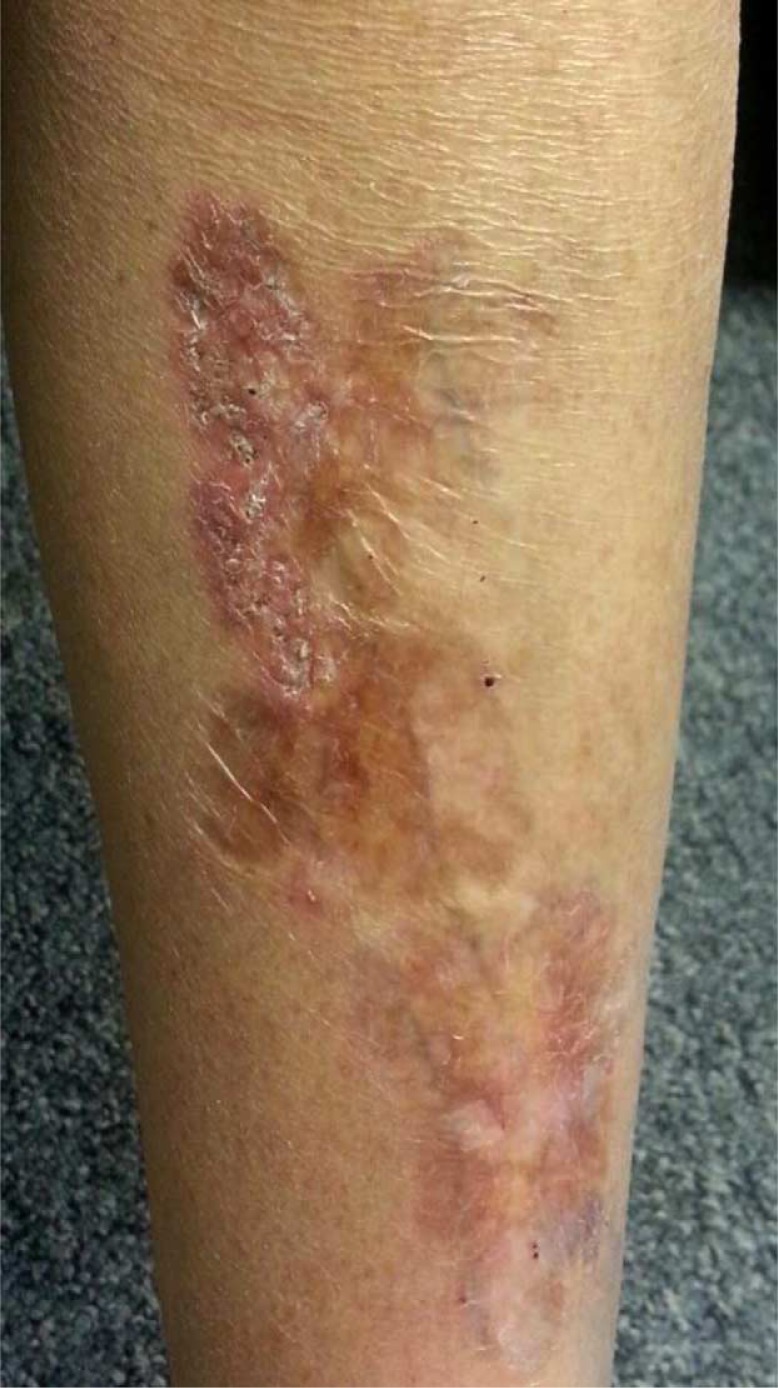
Necrobiosis lipoidica.

Necrobiosis lipoidica is an inflammatory skin disorder of unknown cause, occurring three times more often in females than in males (1). It typically involves the shins, ankles, and feet. It occurs in less than 1% of all diabetic patients (2). It occurs at an earlier age in patients with type 1 DMthan those with type 2 DMor non-diabetics (3). No clear correlation with glycemic control or other complications of DM has been established (4). Although there is no proven treatment for necrobiosis lipoidica, topical, and intra-lesional steroids can be tried for the initial management (5) (Fig. 1). Other agents like tacrolimus, antimalarials, cyclosporine, granulocyte-macrophage colony stimulating factor, hyperbaric oxygen, and systemic corticosteroids have been used with variable results.
